# Early experience of laparoscopic resection and comparison with open surgery for gastric gastrointestinal stromal tumor: a multicenter retrospective study

**DOI:** 10.1038/s41598-022-05044-x

**Published:** 2022-02-10

**Authors:** Shin-Hoo Park, Hyuk-Joon Lee, Min-Chan Kim, Jeong-Hwan Yook, Tae-Sung Sohn, Woo-Jin Hyung, Seung-Wan Ryu, Yukinori Kurokawa, Young-Woo Kim, Sang-Uk Han, Hyung-Ho Kim, Do-Joong Park, Wook Kim, Sang-Il Lee, Haruhiko Cho, Gyu-Seok Cho, Jin-Jo Kim, Ki-Han Kim, Moon-Won Yoo, Han-Kwang Yang

**Affiliations:** 1 Department of Surgery, Seoul National University College of Medicine, 101 Daehak-ro, Jongno-gu, Seoul, 03080 Korea; 2 Department of Surgery, Seoul National University Hospital, Seoul, Korea; 3 Cancer Research Institute, Seoul National University College of Medicine, 101 Daehak-ro, Jongno-gu, Seoul, 03080 Korea; 4 Department of Surgery, Dong-A University College of Medicine, Busan, Korea; 5 Department of Surgery, University of Ulsan College of Medicine, Seoul, Korea; 6 Department of Surgery, Sungkyunkwan University School of Medicine, Samsung Medical Center, Seoul, Korea; 7 Department of Surgery, Yonsei University Health System, Yonsei University College of Medicine, Seoul, Korea; 8 Department of Surgery, Keimyung University School of Medicine, Seoul, Korea; 9 Department of Gastroenterological Surgery, Graduate School of Medicine, Osaka University, Osaka, Japan; 10 Center for Gastric Cancer, National Cancer Center, Seoul, Korea; 11 Department of Surgery, School of Medicine, Ajou University, Suwon, Korea; 12 Department of Surgery, Seoul National University Bundang Hospital, Seoul, Korea; 13 Department of Surgery, Yeouido St. Mary’s Hospital, College of Medicine, The Catholic University of Korea, Seoul, Korea; 14 Department of Surgery, Chungnam National University Hospital, Seoul, Korea; 15 Department of Gastrointestinal Surgery, Kanagawa Cancer Center, Yokohama, Japan; 16 Department of Surgery, Tokyo Metropolitan Cancer and Infectious Diseases Center, Komagome Hospital, Tokyo, Japan; 17 Department of Surgery, Soonchunhyang University College of Medicine, Seoul, Korea; 18 Division of Gastrointestinal Surgery, Department of Surgery, Incheon St. Mary’s Hospital, The Catholic University of Korea, Seoul, Korea

**Keywords:** Gastrointestinal cancer, Surgical oncology

## Abstract

The advantages of laparoscopic resection over open surgery in the treatment of gastric gastrointestinal stromal tumor (GIST) are not conclusive. This study aimed to evaluate the postoperative and oncologic outcome of laparoscopic resection for gastric GIST, compared to open surgery. We retrospectively reviewed the prospectively collected database of 1019 patients with gastric GIST after surgical resection at 13 Korean and 2 Japanese institutions. The surgical and oncologic outcomes were compared between laparoscopic and open group, through 1:1 propensity score matching (PSM). The laparoscopic group (N = 318) had a lower rate of overall complications (3.5% vs. 7.9%, *P* = 0.024) and wound complications (0.6% vs. 3.1%, *P* = 0.037), shorter hospitalization days (6.68 ± 4.99 vs. 8.79 ± 6.50, *P* < 0.001) than the open group (N = 318). The superiority of the laparoscopic approach was also demonstrated in patients with tumors larger than 5 cm, and at unfavorable locations. The recurrence-free survival was not different between the two groups, regardless of tumor size, locational favorableness, and risk classifications. Cox regression analysis revealed that tumor size larger than 5 cm, higher mitotic count, R1 resection, and tumor rupture during surgery were independent risk factors for recurrence. Laparoscopic surgery provides lower rates of complications and shorter hospitalizations for patients with gastric GIST than open surgery.

## Introduction

Gastrointestinal stromal tumors (GISTs) are the commonest type of mesenchymal tumors of the gastrointestinal tract, with the stomach (50–60%) being the most frequent primary site^1^. Although tyrosine kinase inhibitors (TKIs) have significantly improved disease-free survival, complete R0 surgical resection remains the standard treatment for primary non-metastatic gastric GIST^2–4^. Laparoscopic surgery for gastric GIST has been widely performed to meet the current demand for minimal invasiveness. Numerous studies have reported the potential advantages of laparoscopic resection for gastric GISTs, such as earlier return of bowel function, less blood loss, and shorter hospitalization days^5,6^.

However, the perceived advantages of laparoscopic resection compared with open surgery is not conclusive in the treatment of gastric GIST. Previous studies have lacked reliability and credibility for the following reasons. Since gastric GISTs are rare, with an estimated incidence of 1.5/100,000 per year^7,8^, no randomized controlled trial (RCT) has clearly demonstrated the beneficial effect of laparoscopic resection of gastric GIST^9,10^. Therefore, the meta-analyses to date are based on the data extracted from non-RCTs; thus, they have less powerful outcomes^11–14^. In addition, retrospective observational studies that reported similar or even better postoperative laparoscopy results suffer from selection bias since laparoscopic surgeries are preferred for smaller sized tumors with favorable locations^6,15^. Several retrospective studies using propensity score matching (PSM) have been conducted to overcome these limitations. However, the small sample sizes have not been sufficient for clear conclusions, and subgroup analyses were not conducted for patients with tumors larger than 5 cm and in unfavorable locations, simultaneously^16,17^. Although, Xiong et al.^18^ analyzed 1,027 patients, the largest number ever, only 128 patients were remained for analysis in each group after PSM.

Considering the technical advances in laparoscopic surgery over decades, it is meaningful to prove whether laparoscopic surgery was superior to open resection in gastric GISTs treatment even in the early era of laparoscopic surgery. This multi-center retrospective study enrolled 1019 patients diagnosed with gastric GIST after surgery during the period 2000–2007 at 13 Korean and 2 Japanese institutions, aimed to evaluate whether the laparoscopic resections was superior clinical outcomes and comparable in oncologic safety compared to open surgery, even in the early era of laparoscopic surgery. We also evaluated whether the application of laparoscopic surgery could be feasible and safe in tumors larger than 5 cm and in unfavorable locations.

## Results

Before PSM, the laparoscopic group had more patients with a higher propensity score, younger age, tumors ≤ 5 cm, and tumors in a favorable location than the open group. The open group had more cases of gastrectomy, combined resection, higher mitotic count, high-risk by NIH classification, and adjuvant chemotherapy than the laparoscopic group. Except for the missing data, there was no difference between the two groups in immunohistochemical parameters including CD117 and CD34.

After 1:1 PSM, the 318 patients in the laparoscopic group were matched to the 318 patients in the open group in the same period and the distribution of cumulative cases per year has similar distributional tendency with those observed before matching (Supplementary Fig. 1). And, the propensity scores, clinicopathologic variables were all balanced between the laparoscopic and open groups (Table 1). The distribution of the propensity score was more concentrated, and the absolute SMDs of matching covariates decreased near or below 0.1 (Supplementary Fig. 2).

**Table 1:** The clinicopathologic characteristics between the laparoscopic group and open group.

Variables	Before PSM		*P* value	After PSM		*P* value
	Laparoscopic group (n = 373)	Open group (n = 542)		Laparoscopic group (n = 318)	Open group (n = 318)	
**Propensity scores**						
	0.5 ± 0.1	0.3 ± 0.2	< 0.001	0.5 ± 0.2	0.5 ± 0.2	0.056
**Age, years (mean ± SD)**						
	57.1 ± 11.4	60.0 ± 12.0	0.019	57.4 ± 11.3	58.4 ± 11.3	0.245
**Sex**						
Male	175 (46.9)	272 (50.2)	0.346	148 (46.5)	150 (47.2)	0.937
Female	198 (53.1)	270 (49.8)		170 (53.5)	168 (52.8)	
**BMI, kg/m**^**2**^** (mean ± SD)**						
	24.3 ± 3.1	24.0 ± 3.1	0.179	24.2 ± 3.0	24.2 ± 3.1	0.859
**Underlying disease**						
Hypertension	98 (26.4)	170 (31.2)	0.121	84 (26.4)	103 (32.4)	0.117
Cardiovascular	17 (4.6)	23 (4.2)	0.870	13 (4.1)	15 (4.7)	0.847
Cerebrovascular	3 (0.8)	3 (0.6)	0.691	2 (0.6)	1 (0.3)	0.563
Pulmonary disease	19 (5.1)	26 (4.8)	0.877	15 (4.7)	19 (6.0)	0.598
Hepatic disease	22 (5.9)	25 (4.6)	0.366	27 (8.5)	36 (11.3)	0.288
Diabetes mellitus	30 (8.1)	64 (11.8)	0.077	19 (6.0)	17 (5.3)	0.864
Renal disease	2 (0.5)	1 (0.2)	0.569	2 (0.6)	1 (0.3)	0.563
**Radicality of resection**						
R0 resection	368 (98.7)	533 (98.3)	0.790	313 (98.4)	313 (98.4)	1.000
R1 resection	5 (1.3)	9 (1.7)		5 (1.6)	5 (1.6)	
**Extent of resection**						
Wedge resection	343 (92.0)	370 (68.3)	< 0.001	291 (91.5)	288 (90.6)	0.950
Partial gastrectomy	21 (5.6)	88 (16.2)		21 (6.6)	22 (6.9)	
Total gastrectomy	2 (0.5)	78 (14.4)		2 (0.6)	3 (0.9)	
Enucleation	7 (1.9)	6 (1.1)		4 (1.3)	5 (1.6)	
**Combined resection**						
Total	29 (7.8)	83 (15.3)	< 0.001	27 (8.5)	20 (6.3)	0.363
Cholecystectomy	23 (6.2)	19 (3.5)		21 (6.6)	12 (3.8)	
Liver resection	2 (0.5)	5 (0.9)		2 (0.5)	1 (0.3)	
Colon resection	1 (0.3)	17 (3.1)		1 (0.3)	2 (0.5)	
Adrenalectomy	1 (0.3)	7 (1.3)		1 (0.3)	1 (0.3)	
SB resection	1 (0.3)	3 (0.6)		1 (0.3)	1 (0.3)	
Splenectomy	0 (0)	40 (7.4)		0 (0)	1 (0.3)	
Pancreatectomy	0 (0)	20 (3.7)		0 (0)	1 (0.3)	
Others	3 (0.8)	1 (0.3)		3 (0.8)	1 (0.3)	
**Tumor size, mean (mm)**						
	37.3 ± 18.6	65.2 ± 46.5	< 0.001	38.3 ± 19.5	40.1 ± 17.0	0.217
**Tumor size category (cm)**						
> 0, ≤ 2	62 (16.6)	42 (7.7)	< 0.001	52 (16.4)	38 (11.9)	0.554
> 2, ≤ 5	247 (66.2)	241 (44.5)		202 (63.5)	206 (64.8)	
> 5, ≤ 7	49 (13.1)	96 (17.7)		49 (15.4)	58 (18.2)	
> 7, ≤ 10	10 (2.7)	67 (12.4)		10 (3.1)	11 (3.5)	
> 10, ≤ 15	5 (1.3)	69 (12.7)		5 (1.6)	5 (1.6)	
> 15, ≤ 20	0 (0)	17 (3.1)		0 (0)	0 (0)	
> 20, ≤ 30	0 (0)	9 (1.7)		0 (0)	0 (0)	
> 30	0 (0)	1 (0.2)		0 (0)	0 (0)	
**Longitudinal location**						
GEJ to Cardia	59 (15.8)	81 (14.9)	0.540	53 (16.7)	40 (12.6)	0.417
Upper third	153 (41.0)	234 (43.2)		129 (40.6)	146 (45.9)	
Upper to middle	1 (0.3)	2 (0.4)		1 (0.3)	0 (0)	
Middle third	67 (18.0)	96 (17.7)		57 (17.9)	47 (14.8)	
Middle to lower	0 (0)	5 (0.9)		1 (0.3)	1 (0.3)	
Lower third	93 (24.9)	123 (22.7)		77 (24.2)	83 (26.1)	
Entire length	0 (0)	1 (0.2)		0 (0)	1 (0.3)	
**Circumferential location**						
Greater	110 (29.5)	141 (26.0)	0.001	96 (30.2)	89 (28.0)	0.231
Lesser	80 (21.4)	139 (25.6)		68 (21.4)	78 (24.5)	
Anterior	93 (24.9)	90 (16.6)		74 (23.3)	58 (18.2)	
Posterior	90 (24.1)	163 (30.1)		80 (25.2)	91 (28.6)	
Entire	0 (0)	9 (1.7)		0 (0)	2 (0.6)	
**Locational preference**						
Favorable	176 (47.2)	203 (37.5)	0.004	145 (45.6)	130 (40.9)	0.262
Unfavorable	197 (52.8)	339 (62.5)		173 (54.4)	188 (59.1)	
**Mitotic rate (per 50 HPF)**						
≤ 5	276 (74.0)	352 (64.9)	< 0.001	242 (76.1)	242 (76.1)	0.225
> 5, ≤ 10	71 (19.0)	96 (17.7)		56 (17.6)	46 (14.5)	
> 10	26 (7.0)	94 (17.3)		20 (6.3)	30 (9.4)	
**NIH risk stratification**						
Very low	49 (13.1)	36 (6.6)	< 0.001	41 (12.9)	32 (10.1)	0.232
Low	183 (49.1)	183 (33.8)		157 (49.4)	160 (50.3)	
Intermediate	100 (26.8)	128 (23.6)		85 (26.7)	76 (23.9)	
High	41 (11.0)	195 (36.0)		35 (11.0)	50 (15.7)	
**CD 117**						
Yes	306 (97.1)	366 (98.4)	0.303	260 (96.7)	202 (99.0)	0.125
No	9 (2.9)	6 (1.6)		9 (3.3)	2 (1.0)	
Unidentified	58 cases	170 cases		49 case	114 case	
**CD 34**						
Yes	281 (98.6)	333 (97.9)	0.762	241 (98.8)	186 (98.4)	0.752
No	4 (1.4)	7 (2.1)		3 (1.2)	3 (1.6)	
Unidentified	88 case	202 case		74 case	129 case	
**Adjuvant treatment (Gleevec)**						
Yes	7 (1.9)	52 (9.6)	< 0.001	7 (2.2)	7 (2.2)	1.000
No	366 (98.1)	490 (90.4)		311 (97.8)	311 (97.8)	

*GEJ* gastroesophageal junction, *HPF* high power field, *PSM* propensity score matching, *SD* standard deviation, *SB* small bowel.

### Surgical outcomes and complications before and after PSM

The rates of overall complications were significantly lower in the laparoscopic group than in the open group, either before (4.0% vs. 8.3%, *P* = 0.010) or after PSM (3.5% vs. 7.9%, *P* = 0.024). The complication requiring major intervention were lower in the laparoscopic group than in the open group, either before (2.1% vs. 5.5%, *P* = 0.011) or after PSM (1.9% vs. 5.7%, *P* = 0.020). The laparoscopic group had lower wound complications than the open group (0.6% vs. 3.1%, *P* = 0.037) after PSM. Intestinal motility disorder was not found in the laparoscopic group but was found in the open group. The hospitalization days of the laparoscopic group were significantly shorter than those of the open group, either before (6.7 ± 4.9 vs. 10.2 ± 7.7, *P* < 0.001) or after PSM (6.7 ± 5.0 vs. 8.8 ± 6.5, *P* < 0.001). The mortality rate within 30 days was not different between the two groups, regardless of PSM (Table 2).

**Table 2:** Operative outcomes and surgical complications of gastric GIST patients treated by laparoscopic versus open resection.

Variable	Before PSM		*P* value	After PSM		*P* value
	Laparoscopic group (N = 373)	Open group (N = 542)		Laparoscopic group (N = 318)	Open group (N = 318)	
**Operation time (minute)**						
Overall	113.9 ± 58.6	125.5 ± 71.3	0.011	114.0 ± 57.0	100.8 ± 54.5	0.003
Wedge resection or enucleation	112.0 ± 58.6	103.4 ± 59.7	0.052	111.7 ± 56.9	97.4 ± 53.7	0.002
Gastrectomy	143.7 ± 51.1	175.4 ± 70.2	0.014	143.1 ± 50.3	143.6 ± 46.3	0.973
**Conversion to open surgery**	14 (3.8)	–	–	11 (3.5)	–	–
**Tumor rupture during surgery**	0 (0)	13 (2.4)	0.001	0 (0)	6 (1.9)	0.031
**Hospital days (mean ± SD)**						
Overall	6.7 ± 4.9	10.1 ± 7.7	< 0.001	6.7 ± 5.0	8.8 ± 6.5	< 0.001
Wedge resection or enucleation	6.5 ± 4.8	9.2 ± 7.8	< 0.001	6.4 ± 4.9	8.7 ± 6.7	< 0.001
Gastrectomy	9.8 ± 5.6	12.0 ± 7.2	0.094	8.8 ± 2.6	9.4 ± 2.3	0.389
**Postoperative complications**						
Total number of cases with complication	15 (4.0)	45 (8.3)	0.010	11 (3.5)	25 (7.9)	0.024
Wound	4 (1.1)	13 (2.4)	0.212	2 (0.6)	10 (3.1)	0.037
Fluid collection	1 (0.3)	6 (1.1)	0.251	1 (0.3)	1 (0.3)	1.000
Intraabdominal bleeding	3 (0.8)	6 (1.1)	0.746	3 (0.9)	3 (0.9)	1.000
Luminal bleeding	3 (0.8)	2 (0.4)	0.403	2 (0.6)	1 (0.3)	0.563
Stenosis	2 (0.5)	4 (0.7)	0.718	2 (0.7)	1 (0.3)	0.563
Intestinal motility disorder	0 (0)	6 (1.1)	0.087	0 (0)	5 (1.6)	0.062
Leakage	1 (0.3)	2 (0.4)	0.799	0 (0)	1 (0.3)	0.317
Fistula	0 (0)	2 (0.4)	0.517	0 (0)	1 (0.3)	0.317
Pulmonary complications	0 (0)	5 (0.9)	0.085	0 (0)	4 (1.3)	0.124
Urinary complications	1 (0.3)	2 (0.4)	0.793	1 (0.3)	1 (0.3)	1.000
Hepatic complications	0 (0)	2 (0.4)	0.517	0 (0)	0 (0)	–
**Clavien–Dindo classification**						
Grade I	3 (0.3)	6 (1.1)	0.745	2 (0.6)	4 (1.3)	0.686
Grade II	4 (1.1)	14 (2.6)	0.146	3 (0.9)	6 (1.9)	0.505
Grade IIIa	8 (2.1)	17 (3.1)	0.415	6 (1.9)	8 (2.5)	0.788
Grade IIIb	0 (0)	10 (1.8)	0.007	0 (0)	8 (2.5)	0.007
Grade IVa	0 (0)	1 (0.2)	0.407	0 (0)	0 (0)	–
Grade IVb	0 (0)	0 (0)	–	0 (0)	0 (0)	–
Grade V	0 (0)	2 (0.4)	0.517	0 (0)	2 (0.6)	0.499
Complication requiring major intervention (≥ grade IIIa)	8 (2.1)	30 (5.5)	0.011	6 (1.9)	18 (5.7)	0.020
**Mortality within 30 days**	0 (0)	2 (0.4)	0.517	0 (0)	2 (0.6)	0.499

*PSM* propensity score matching, *SD* standard deviation.

The operation time was shorter in the laparoscopic group than in the open group before PSM (113.9 ± 58.6 min vs. 125.5 ± 71.3 min, *P* = 0.011). After PSM, the operation time of the laparoscopic group was longer than that of the open group (114.0 ± 57.0 min vs. 100.8 ± 54.5 min, *P* = 0.003) (Table 2).

Regardless of PSM, tumor rupture during surgery was not detected in the laparoscopic group. The laparoscopic group showed a significantly lower rate of tumor rupture during surgery than the open group, either before or after PSM (all *P* < 0.05). Before PSM, 13 patients experienced tumor rupture during open surgery (Table 2).

Of the 373 patients who were initially planned for laparoscopic surgery, 14 patients (3.8%) required conversion to open surgery. Detailed explanations for the reason of each case that required conversion to open surgery were described in Supplementary Table 1. The favorable or unfavorable location did not determine the conversion, regardless of PSM. However, the conversion rate was significantly higher in tumors > 5 cm than in those < 5 cm, either before or after PSM (all *P* < 0.05) (Supplementary Table 2).

### Surgical outcomes and complications by subgroup analysis

Multivariate logistic regression analysis revealed that open resection, longer operation time, tumor size larger than 5 cm, and unfavorable locations were independent risk factors for overall complications (Supplementary Table 3). Because, the rate of overall complications can vary depending on the tumor size and locational preferences, subgroup analyses for surgical outcomes including complications were performed between laparoscopic versus open resection, according to the tumor size and locational preferences.

Regarding the subgroup analysis according to tumor size, the complication rates were not different in patients with tumors ≤ 5 cm between the two groups, but the laparoscopic group had a lower rate of overall (3.1% vs. 16.2%, *P* = 0.012) and major complication (1.6% vs. 12.2%, *P* = 0.020) than the open group, in patients with tumors > 5 cm. The hospitalization days were shorter in the laparoscopic group than in the open group, either in patients with tumors ≤ 5 cm or > 5 cm (all *P* < 0.05, Table 3).

**Table 3:** Operative outcomes and surgical complications of patients with gastric GISTs according to tumor size.

Variable	Tumor size ≤ 5 cm		*P* value	Tumor size > 5 cm		*P* value
	Laparoscopic group (N = 254)	Open group (N = 244)		Laparoscopic group (N = 64)	Open group (N = 74)	
**Operation time (minute)**						
Overall	112.5 ± 58.9	100.0 ± 53.3	0.015	120.1 ± 48.8	103.5 ± 58.6	0.073
**Conversion to open surgery**	5 (2.0)			6 (9.4)	–	–
**Tumor rupture during surgery**	0 (0)	3 (1.2)	0.117	0 (0)	3 (4.1)	0.248
**Hospital days (mean ± SD)**						
Overall	6.5 ± 4.2	8.8 ± 6.7	< 0.001	6.6 ± 2.7	9.0 ± 5.9	0.003
**Postoperative complications**						
Total number of cases with complication	9 (3.5)	13 (5.3)	0.387	2 (3.1)	12 (16.2)	0.012
Wound	1 (0.4)	5 (2.0)	0.116	1 (1.6)	5 (6.8)	0.216
Fluid collection	1 (0.4)	0 (0)	0.327	0 (0)	1 (1.4)	0.351
Intraabdominal bleeding	3 (1.2)	3 (1.2)	0.961	0 (0)	0 (0)	–
Luminal bleeding	1 (0.4)	1 (0.4)	0.977	1 (1.6)	0 (0)	0.464
Stenosis	2 (0.9)	1 (0.5)	0.600	0 (0)	0 (0)	–
Intestinal motility disorder	0 (0)	2 (0.8)	0.240	0 (0)	3 (4.1)	0.248
Leakage	0 (0)	1 (0.4)	0.490	0 (0)	0 (0)	–
Fistula	0 (0)	0 (0)	–	0 (0)	1 (1.4)	0.351
Pulmonary complications	0 (0)	2 (0.8)	0.240	0 (0)	2 (2.7)	0.499
Urinary complications	1 (0.4)	0 (0)	0.327	0 (0)	1 (1.4)	0.351
**Clavien–Dindo classification**						
Grade I	1 (0.4)	3 (1.2)	0.364	1 (1.6)	1 (1.4)	0.918
Grade II	3 (1.2)	3 (1.2)	1.000	0 (0)	3 (4.1)	0.248
Grade IIIa	5 (2.0)	4 (1.6)	0.783	1 (1.6)	4 (5.4)	0.373
Grade IIIb	0 (0)	4 (1.6)	0.057	0 (0)	4 (5.4)	0.123
Grade IVa	0 (0)	0 (0)	–	0 (0)	0 (0)	–
Grade IVb	0 (0)	0 (0)	–	0 (0)	0 (0)	–
Grade V	0 (0)	1 (0.4)	0.490	0 (0)	1 (1.4)	0.351
Complication requiring major intervention (≥ grade IIIa)	5 (2.0)	9 (3.7)	0.287	1 (1.6)	9 (12.2)	0.020
**Mortality within 30 days**	0 (0)	1 (0.4)	0.490	0 (0)	1 (1.4)	0.351

*SD* standard deviation.

According to locational preference, the rate of overall complications was not different between the two groups for tumors in favorable locations. However, the laparoscopic group had a significantly lower rate of complications than the open group for tumors in unfavorable locations (4.0% vs. 10.1%, *P* = 0.040). The hospitalization days of the laparoscopic group were shorter than those of the open group, indiscriminate of locational preference (all *P* < 0.05, Table 4).

**Table 4:** Operative outcomes and surgical complications according to locational preference (favorable versus unfavorable).

Variable	Favorable location		*P* value	Unfavorable location		*P* value
	Laparoscopic group (N = 145)	Open group (N = 130)		Laparoscopic group (N = 173)	Open group (N = 188)	
**Operation time (minute)**						
Overall	113.4 ± 59.6	100.5 ± 59.4	0.079	114.6 ± 54.8	101.1 ± 51.1	0.017
**Conversion to open surgery**	3 (2.1)			8 (4.6)	–	–
**Tumor rupture during surgery**	0 (0)	1 (0.8)	0.473	0 (0)	5 (2.7)	0.062
**Hospital days (mean ± SD)**						
Overall	6.7 ± 5.2	8.6 ± 5.7	0.006	6.6 ± 4.8	8.9 ± 7.0	< 0.001
**Postoperative complications**						
Total number of cases with complication	4 (2.8)	6 (4.6)	0.525	7 (4.0)	19 (10.1)	0.040
Wound	1 (0.7)	3 (2.3)	0.347	1 (0.6)	7 (3.7)	0.069
Fluid collection	1 (0.7)	0 (0)	0.343	0 (0)	1 (0.5)	0.337
Intraabdominal bleeding	0 (0)	1 (0.8)	0.473	3 (1.7)	2 (1.1)	0.674
Luminal bleeding	1 (0.7)	1 (0.8)	0.938	1 (0.6)	0 (0)	0.479
Stenosis	0 (0)	0 (0)	–	2 (1.2)	1 (0.5)	0.609
Intestinal motility disorder	0 (0)	0 (0)	–	0 (0)	5 (2.7)	0.062
Leakage	0 (0)	0 (0)	–	0 (0)	1 (0.5)	0.337
Fistula	0 (0)	1 (0.8)	0.473	0 (0)	0 (0)	–
Pulmonary complications	0 (0)	1 (0.8)	0.473	0 (0)	3 (1.6)	0.249
Urinary complications	1 (0.7)	1 (0.8)	0.938	0 (0)	0 (0)	–
**Clavien–Dindo classification**						
Grade I	1 (0.7)	3 (2.3)	0.347	1 (0.6)	1 (0.5)	0.953
Grade II	2 (1.4)	1 (0.8)	0.627	1 (0.6)	5 (2.7)	0.217
Grade IIIa	1 (0.7)	3 (2.3)	0.347	5 (2.9)	6 (3.2)	0.868
Grade IIIb	0 (0)	0 (0)	–	0 (0)	7 (3.7)	0.015
Grade IVa	0 (0)	0 (0)	–	0 (0)	0 (0)	–
Grade IVb	0 (0)	0 (0)	–	0 (0)	0 (0)	–
Grade V	0 (0)	1 (0.8)	0.473	0 (0)	1 (0.5)	0.337
Complication requiring major intervention (grade ≥ IIIa)	1 (0.7)	4 (3.1)	0.192	5 (2.9)	14 (7.4)	0.061
**Mortality within 30 days**	0 (0)	1 (0.8)	0.473	0 (0)	1 (0.5)	0.337

*SD* standard deviation.

### Oncologic outcomes

**Figure 1:**
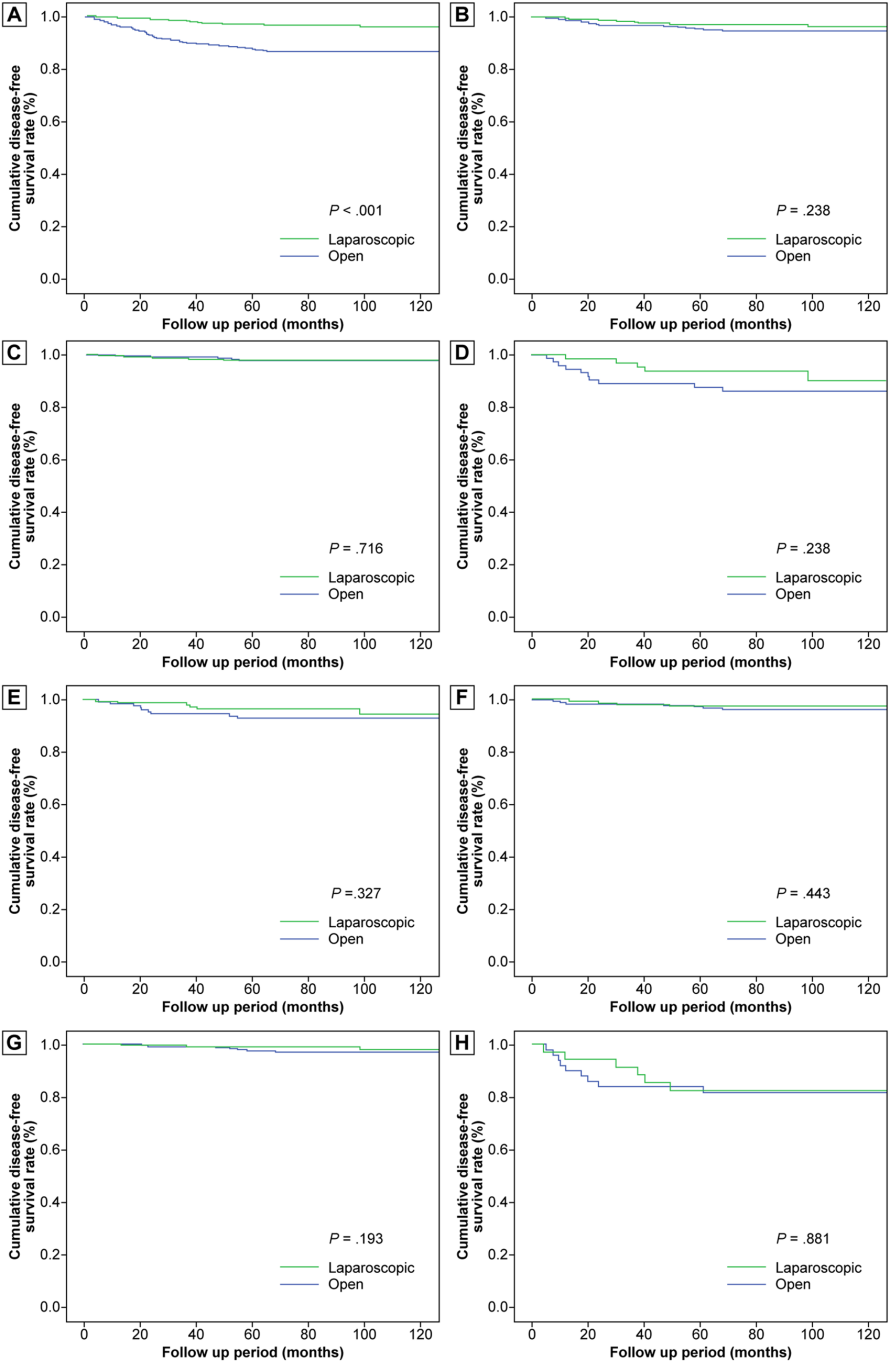
Comparison of disease-free survival between the laparoscopic and open groups. (**a**) Disease-free survival in all patients, before 1:1 propensity score matching. (**b**) Disease-free survival in all patients, after 1:1 propensity score matching. (**c**) Disease-free survival in patients with tumor ≤ 5 cm, after 1:1 propensity score matching. (**d**) Disease-free survival in patients with tumor > 5 cm, after 1:1 propensity score matching. (**e**) Disease-free survival in patients with tumor at favorable location, after 1:1 propensity score matching. (**f**) Disease-free survival in patients with tumor at unfavorable location, after 1:1 propensity score matching. (**g**) Disease-free survival in patients with very low to intermediate risk by National Institutes of Health classification, after 1:1 propensity score matching. (**h**) Disease-free survival in patients with high risk by National Institutes of Health classification, after 1:1 propensity score matching.

Before PSM, the open group had more patients with adjuvant chemotherapy, longer follow-up duration, recurrence, and death. The recurrence-free survival rates of the laparoscopic group were superior to those of the open groups (all *P* < 0.05). After PSM, the parameters related to oncologic outcomes were not different between the two groups. The recurrence-free survival rates were not different between the two groups, regardless of tumor size, locational preference, and NIH risk classification (all *P* > 0.05) (Table 5, Fig. 1).

**Table 5:** Oncologic outcomes for laparoscopic versus open resection before and after propensity score matching.

Variables	Before PSM		*P* value	After PSM		*P* value
	Laparoscopic group (N = 373)	Open group (N = 542)		Laparoscopic group (N = 318)	Open group (N = 318)	
**Chemotherapy**						
Preoperative	1 (0.3)	6 (1.1)	0.251	1 (0.3)	2 (0.6)	0.563
Adjuvant	7 (1.9)	52 (9.6)	< 0.001	7 (2.2)	7 (2.2)	0.000
**Duration of follow-up**						
Mean	93.6	103.5	< 0.001	95.1	98.8	0.114
Median (range)	88.0 (11.0–164.7)	106.4 (0.1–167.9)		88.5 (25.2–164.7)	100.7 (0.1–144.2)	
**Recurrence**						
Total number	13 (3.5)	72(13.3)	< 0.001	10 (3.1)	16 (5.0)	0.317
Stomach	5 (1.3)	16 (3.0)	0.121	5 (1.6)	3 (0.9)	0.725
Small bowel	0 (0)	1 (0.2)	0.407	0 (0)	1 (0.3)	0.317
Liver	2 (0.5)	36 (6.6)	< 0.001	1 (0.3)	7 (2.2)	0.069
Peritoneum	5 (1.3)	20 (3.7)	0.038	4 (1.3)	2 (0.6)	0.686
Bone	0 (0)	3 (0.6)	0.275	0 (0)	0 (0)	–
Wound	0 (0)	1 (0.3)	0.408	0 (0)	1 (0.3)	0.317
Lung	0 (0)	1 (0.2)	0.407	0 (0)	1 (0.3)	0.317
Axilla	1 (0.3)	3 (0.6)	0.649	1 (0.3)	2 (0.6)	0.563
**Alive**						
Total number	365 (97.9)	499 (92.1)	< 0.001	311 (97.8)	307 (96.5)	0.474
with recurrence free	355 (95.2)	448 (82.7)	< 0.001	303 (95.3)	296 (93.1)	0.173
with recurrence	10 (2.7)	51 (9.4)	< 0.001	8 (2.5)	11 (3.5)	0.643
**Death**						
Total number	8 (2.1)	43 (7.9)	< 0.001	7 (2.2)	11 (3.5)	0.474
Died due to GIST	3 (0.8)	21 (3.9)	0.005	2 (0.6)	5 (1.6)	0.451
Died of other causes	5 (1.3)	22 (4.1)	0.017	5 (1.6)	6 (1.9)	0.761

*GIST* gastrointestinal stromal tumor, *PSM* propensity score matching.

Multivariate Cox regression analysis revealed the following independent risk factors: R1 (vs. R0) resection (hazard ratio [HR] = 6.327, 95% confidence interval [CI] = 1.826–21.919, *P* = 0.004), tumor size > 5 cm (vs. ≤ 5 cm) (HR = 3.080, 95% CI = 1.368–6.934, *P* = 0.007), mitotic count > 5, ≤ 10 (vs. ≤ 5) (HR = 8.090, 95% CI = 2.651–24.685, *P* < 0.001), mitotic count > 10 (vs. ≤ 5) (HR = 32.666, 95% CI = 10.586–100.798, *P* < 0.001), and tumor rupture during surgery (HR = 63.479, 95% CI = 17.407–231.488, *P* < 0.001) (Table 6**)**.

**Table 6:** Univariate and multivariate Cox regression analysis for variables to predict the recurrence.

Variables	Univariate Cox regression			Multivariate Cox regression		
	B	Hazard ratio (95% CI)	*P* value	B	Hazard ratio (95% CI)	*P* value
**Age, years per increase**	0.006	1.006 (0.972–1.041)	0.740			
**Male** (vs. female)	0.461	1.586 (0.728–3.453)	0.245			
**Body mass index** (kg/m^2^, per increase)	− 0.069	0.934 (0.821–1.062)	0.296			
**Laparoscopy** (vs. open)	− 0.472	0.624 (0.283–1.375)	0.242			
**Extent of resection**						
Gastrectomy versus wedge or enucleation	1.401	4.059 (1.629–10.116)	0.003			
**Radicality, R1** (vs. R0 resection)	2.146	8.550 (2.567–28.484)	< 0.001	1.845	6.327 (1.826–21.919)	0.004
**Adjuvant therapy**	2.238	9.377 (3.226–27.259)	< 0.001			
**Tumor size**						
> 5 cm (vs. ≤ 5 cm)	1.626	5.082 (2.334–11.064)	< 0.001	1.125	3.080 (1.368–6.934)	0.007
**Mitotic rate (per 50 HPF)**						
> 5, ≤ 10 (vs. ≤ 5)	1.913	6.771 (2.348–19.524)	< 0.001	2.091	8.090 (2.651–24.685)	< 0.001
> 10 (vs. ≤ 5)	3.135	22.983 (8.617–61.298)	< 0.001	3.486	32.666 (10.586–100.798)	< 0.001
**NIH risk classification**						
High (vs. very low to intermediate)	2.457	11.664 (5.290–25.721)	< 0.001			
**Tumor rupture during surgery**	3.208	24.724 (8.467–72.190)	< 0.001	4.151	63.479 (17.407–231.488)	< 0.001

Covariates were age, sex, body mass index (kg/m^2^), laparoscopic approach, extent of resection, radicality, adjuvant therapy, tumor size, mitotic rate, NIH risk classification, tumor rupture during surgery.

*CI* confidence interval, *HPF* high power field, *NIH* National institutes of health.

## Discussion

This study reviewed the multicentric database from 13 institutions in Korea and 2 in Japan, with more than 1019 patients who underwent laparoscopic and open resection for primary gastric GIST. Long-term surgical and oncologic outcomes for the treatment of primary gastric GIST have been reported based on this retrospective data^19^, and this is a subsequent, collateral study comparing the outcomes between laparoscopic and open surgery. Since resection of gastric GISTs is simple and does not require extensive lymphadenectomy with wide tumor-free margins^20,21^, the benefits of laparoscopic surgery for gastric GIST may not be as obvious as in gastric cancer surgery. Moreover, while Korean Laparo-endoscopic Gastrointestinal Surgery Study Group (KLASS) RCTs proved superiority of laparoscopic over open gastrectomy, no other RCTs have demonstrated the beneficial effect of laparoscopic resection for gastric GIST. Therefore, it seems impatient to generalize that a laparoscopic approach is beneficial for GIST surgery. Even, Xiong et al.^18^ enrolled more patients than current study, they could only compare 128 cases in each group for analysis after PSM. However, the current study remained the largest number of cases after PSM, with more than 300 patients in each group^16,18^. The PSM was carried out in the direction of preserving the sample size as many as possible, by including only four essential variables that may affect the preference of laparoscopic approach and the patient's clinical course. After eliminating the preferential selection bias with PSM, the clinicopathologic characteristics were balanced between the two groups. The distribution of the propensity score was more concentrated, and the absolute SMDs of matching covariates decreased near 0.1. All these efforts contributed to overcoming the bias of a retrospective approach and provided robustness to the current study.

We demonstrated that laparoscopic resection provided superior outcomes, such as shorter hospitalization days, lower rate of tumor rupture, and overall and wound complications than open surgery for the treatment of gastric GIST, even in the early era of the laparoscopic surgery. The patients with tumors larger than 5 cm and in unfavorable locations were also provided with advantages of laparoscopic surgery. Intestinal motility disorder and tumor rupture were not identified in laparoscopic surgery. While the beneficial effects of laparoscopic distal gastrectomy in the KLASS 01 and 02 trials were obtained through the surgeons’ strict quality control^9,10^, the results of the current study are more realistic, as they were based on the data generated by the surgeons’ unrefined experience for laparoscopic surgery. The superior surgical outcome of the laparoscopic approach can be explained by the following reasons. First, the highly magnified view through the laparoscopic camera might enable the surgeon to perform more meticulous dissection, which could avoid unexpected bleeding or tumor rupture^9^. Second, less manipulation of the bowel and smaller wound, might enable patients to have early ambulation and return to eating, with less pain and better peristalsis^22,23^. Third, although the annual experience of laparoscopic surgery differed from one person to another (0–100 cases) in the mid to late 1990s, all surgeons participating in current study had at least 5 years of experiences performing 50–150 cases of open gastrectomy per year prior to initiating laparoscopic gastric surgery. From the year of 2003, even surgeons without any experience in laparoscopic procedures began performing at least 30 cases of laparoscopic surgeries for gastric cancer or GIST every year. Besides, resection of gastric GISTs is simple and does not require extensive lymphadenectomy with wide tumor-free margins as in gastric cancer surgery. These various factors might have allowed to provide an opportunity for better realization of minimal invasiveness, which ultimately resulted in faster recovery and better postoperative outcomes, even in the early era of laparoscopic surgery without surgeon’s quality control.

Nevertheless, laparoscopic approaches have traditionally been recommended for patients with GISTs less than 5 cm, or in favorable locations, according to the NCCN guidelines^24,25^. Huang et al. reported that the unfavorable group had longer hospital stays and more postoperative complications than the favorable group^26^. We also revealed that tumor size larger (vs. less) than 5 cm and unfavorable (vs. favorable) location were independent risk factors for postoperative complications. For these reasons, it is meaningful to investigate whether laparoscopic surgery can lower the rate of surgical complications under these circumstances. Fortunately, successful laparoscopic resection for gastric GISTs larger than 5 cm has been steadily reported with the largest diameters up to 10.5 cm and 11.5 cm^27,28^. Indeed, the postoperative outcomes of laparoscopic surgery were reported to be comparable or even better with tumors larger than 5 cm^29,30^. Regarding the unfavorable locations, the incidence of grade III–IV postoperative complications from laparoscopic surgery was reported to be significantly less than that of open surgery (2.3% vs. 29.0%, *P* = 0.001)^26^. This study also demonstrated that laparoscopic resection provided shorter hospital stays and lower complication rates in patients with tumors larger than 5 cm and at unfavorable locations.

Despite the superior surgical outcome of laparoscopic surgery, surgeons face skepticism that laparoscopic resection also requires laparotomy at least as large as the size of the tumor to retrieve the specimen. In the open surgery, laparotomy larger than tumor size is often necessary to obtain a better view of the invisible side beyond tumor for safe resection. However, laparoscopic camera can reach and visualize the marginal area beyond the large tumor even in unfavorable locations. Laparoscopic surgery only requires a length of incision that fits the tumor size, with the aid of laparoscopic pouch bag. As the laparoscopic procedures become more minimally invasive, the wound length can be shortened to the minimum size, required only for specimen retrieval^4,31^. Although analysis of the incision length was not possible due to the retrospective nature of the current study, we expect that the shorter wound length would have contributed to the lower rate of wound complication in laparoscopic resection than in open surgery, as in previous studies^32^.

Of the 373 cases with laparoscopic resection, 14 cases (3.8%) required conversion to open surgery, lower than that in most reported series (7% [range 0–25])^16,29^. Our study showed that the conversion rate was not related to locational preference. Instead, the conversion rate was higher in patients with tumors larger than 5 cm (Supplementary Table 2). Considering that Kitano et al.^33^ first introduced the laparoscopic technique in the field of gastric cancer surgery in 1994, laparoscopic resection for gastric GIST would not have been actively performed in the early 2000s, which is similar to our results (Supplementary Fig. 2). Moreover, the gap in operation time between the laparoscopic and open groups narrowed and improved from 2006 (108.53 ± 63.43 min. vs. 97.62 ± 46.36 min, *P* = 0.177), compared to the year before 2006 (119.87 ± 48.68 min. vs. 101.94 ± 57.05 min., *P* = 0.001). Recent studies reported a faster operation time in laparoscopic resection than in open surgery for gastric GISTs larger than 5 cm^29^. With advances in laparoscopic techniques, the rate of conversion to open surgery may reduce further.

A superior RFS in laparoscopic resection than in open surgery, can be explained by the differences in the clinical and pathological characteristics, which exert a great impact on the oncologic outcome of GIST^34^. Following our elimination of confounding variables using 1:1 PSM, we showed no significant difference in the recurrence-free survival between the laparoscopic and open groups, even in patients with tumors larger than 5 cm, at unfavorable locations, and in high-risk groups. Several studies including our previous research reported that male sex and non-gastric location were also risk factors for recurrence after resection of gastric GIST^19,20,35^. In the current study, multivariate Cox regression analysis revealed that tumor size > 5 (vs. ≤ 5) cm, higher mitotic count, R1 (vs. R0) resection, and tumor rupture during surgery were independent risk factors for the recurrence of gastric GIST, after eliminating confounding effects by PSM. Laparoscopic or open approaches were not associated with recurrence. The patients with laparoscopic resection and open surgery had similar recurrence and survival rates, by balancing the baseline characteristics of patients after PSM.

This study has some limitations. First, the patients were enrolled from the year 2000, when surgical methods, laparoscopic indications, and pre and postoperative treatment guidelines for gastric GIST were not standardized. Since the worldwide incidence of gastric GIST is rare, it took about 8 years to recruit more than 1000 patients. Additionally, imatinib mesylate (Gleevec) was covered by the Korean health insurance system from June 2001. Therefore, the trends in treatment for gastric GIST may have changed during our study period. Nevertheless, we tried to further highlight the advantages of laparoscopic surgery by demonstrating that laparoscopic approach was superior to open surgery in the treatment of gastric GISTs even in the early 2000s, when laparoscopic techniques were not fully advanced. Second, since we were unable to review the detailed pathologic information of patients prior to the introduction of electrical medical records, the staining information for CD117 and CD34 was not available in some patients; we only included patients with clear medical records who were diagnosed with gastric GIST after surgical resection. In each institution, the diagnosis of gastric GIST was confirmed by reviewing the patient's pathologic findings, which included both typical morphological features and information from IHC staining. We inevitably included patients without information on IHC staining for CD117 and CD34 to preserve as many cases as possible. All patients negative for CD117 were positive for CD34, and the remaining patients with available data on IHC also showed a similar rate of distribution of C-kit positivity as in previous studies^26,36^. Third, detailed information on accidental intraoperative injury to adjacent organs could not be analyzed for similar reasons. Fourth, although the concept of favorable and unfavorable locations had not been established during the period 2000–2007, only patients with clear information about both longitudinal and circumferential locations on the endoscopic and surgical records could be included in this study. Therefore, we were able to classify patients with favorable and unfavorable locations by retrospective reviewing the longitudinal and circumferential locations^24,26^. Fifth, although PSM is a good method to reduce selection bias, it cannot eliminate this bias completely as RCT would be able to. We excluded only the cases with distant metastasis or invasion to adjacent organs, and then matched the variables determining the preferences for the laparoscopic approach, to preserve as many cases as possible. Therefore, similar recurrence and survival rates between laparoscopic resection and open surgery were obtained by balancing baseline characteristics, not due to the washing out effect of PSM. Finally, PSM analysis inevitably excluded the most of cases requiring combined resection and patients with tumors larger than 15 cm, the superiority of laparoscopic surgery could not be analyzed in these population (Table 1). Future studies are expected to provide answer for these issues.

## Conclusions

Even in the early era of laparoscopic surgery with unrefined experiences, laparoscopic resection could provide a lower rate of complications and shorter hospitalization for patients with gastric GIST than open surgery, without compromising oncological safety. Since its first introduction in the early 1990s, laparoscopic gastrectomy has technologically revolutionized the treatment of gastric cancer and almost 30 years have passed. Considering the development of laparoscopic technology over decades, and the current study having large number of cases with more than 300 in each group after 1:1 matching, wider adoption of laparoscopic resection is expected in clinical practice, even in patients with tumor sizes larger than 5 cm and in unfavorable locations.

## Materials and methods

### Study design and cohorts

**Figure 2:**
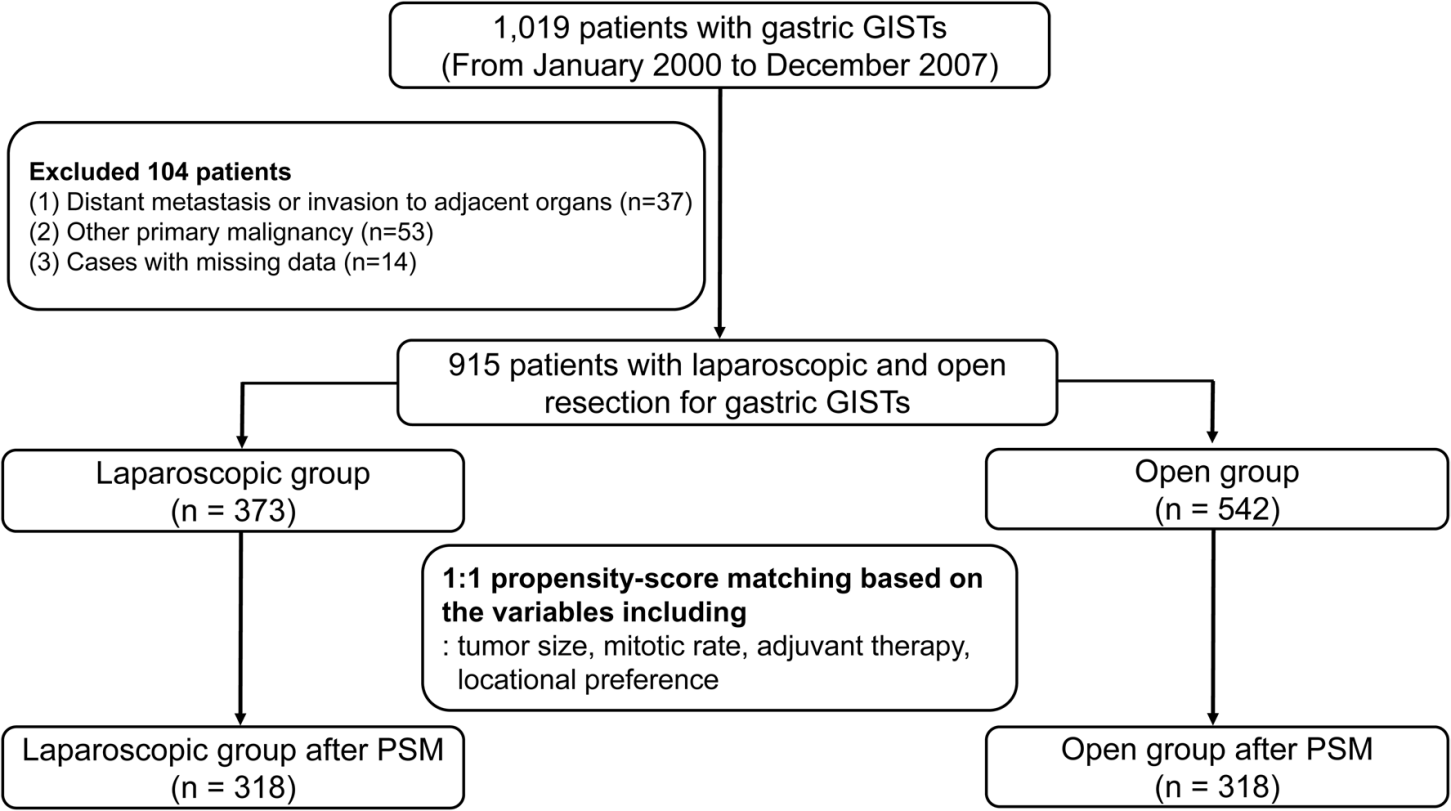
The flow chart for the patient selection model.

From 13 institutions in Korea and 2 institutions in Japan, we retrospectively reviewed the prospectively collected database of 1019 patients who were diagnosed with gastric GIST after surgical resection between January 2000 and December 2007. In each institution, the diagnosis of gastric GIST was confirmed by reviewing the patient's pathologic findings, which included typical morphological features and staining for immunohistochemistry (IHC). The exclusion criteria were as follows: (1) distant metastasis or invasion of the adjacent organs; (2) concomitant other primary malignancy; and (3) cases with missing data (Fig. 2).

The primary outcomes were surgical complications. Secondary outcomes included disease-free survival, rate of recurrence, and survival. Patients were divided into two groups: those who underwent laparoscopic gastric resection (laparoscopic group) and those who underwent open surgery (open group). The prospectively collected clinicopathologic data and operative parameters from each institution were retrospectively reviewed. Mitotic rate was defined as the number of mitoses per 50 high-power fields. Risk stratification was performed according to the modified National Institutes of Health (NIH) risk classification. Although, the concept of favorable and unfavorable locations had not been established during the period 2000–2007, we were able to classify patients according to unfavorable (the gastroesophageal junction, the lesser curvature, the posterior wall, the antrum, and the pylorus) and favorable locations (all locations except the unfavorable locations, which included the greater curvature and anterior wall of the stomach), by retrospectively reviewing endoscopic and operative records with longitudinal and circumferential locations^24,26^. Therefore, only patients with clear information about both longitudinal and circumferential locations on the endoscopic and surgical records were included in this study. Patients were followed up every 6 months or annually, and the follow-up information included adjuvant treatment, survival or death, and recurrences.

This study was approved by the Institutional Review Board (IRB) of Seoul National University Hospital (IRB number: H2006-176-1135) and each participating institution, conducted in accordance with the 1964 Declaration of Helsinki and newer amendments.

### Propensity score matching

Each case from the laparoscopic group was 1:1 propensity score matched to control cases in the open group. The matching variables included tumor size, mitotic rate, locational preference (favorable, or unfavorable), and adjuvant chemotherapy. The propensity score of each patient was estimated by logistic regression (SPSS version 25; IBM Corp., Armonk, NY) and matched nearest-neighbor value within a caliper 0.02 times the standard deviation of the estimated score. After 1:1 PSM, the balance of covariates between the laparoscopic and open groups was measured by calculating the standardized mean difference. A standardized mean difference (SMD) of less than 0.1 was considered to achieve balance and SMD ranging from 0.1 to 0.25 were considered as acceptable disparity between groups^37^.

### Surgical and oncologic outcome

The operative outcomes including surgical complications, operation time, tumor rupture during surgery and hospitalization days were compared between the laparoscopic and open groups, both before and after 1:1 PSM. After performing multivariate logistic regression analysis to reveal the independent factors for surgical complications, we separately collected the patients with such risk factors to determine the presence of complications. We then compared the operative outcomes including surgical complications between the laparoscopic and open groups, for these subgroups. Complication categories were based on the categories used in previous phase III multi-center randomized controlled trials evaluating the complications of laparoscopic gastrectomy. Intestinal motility disorder refers to the peristaltic problem in intestinal tract including mechanical or paralytic ileus, intestinal obstructions^38,39^.

Survival periods were defined as the time from operation to death associated with the GIST or not, which were censored at the final follow-up visit. The recurrence-free survival was calculated as the time from operation to the event of recurrent disease. Recurrence-free survival was compared between the laparoscopic and open groups, either before or after 1:1 PSM. Subgroup analysis for recurrence-free survival was performed according to tumor size, locational preference, and NIH risk classification. Multivariate analysis with Cox proportional hazard regression was used to determine the independent prognostic factors to predict the tumor recurrence.

### Statistical analysis

The chi-square test was used for categorical variables, and Student’ s t-test was used to compare continuous variables between the two groups. All tests were two-sided, and a *P* value of < 0.05 was considered statistically significant. Statistical analyses were performed using IBM SPSS Statistics version 25. The recurrence-free survival was assessed by Kaplan–Meier analysis and the log-rank test.

### Compliance with ethical standards

All procedures followed were in accordance with the ethical standards of the responsible committee on human experimentation (institutional and national) and with the Helsinki Declaration of 1964 and later versions. This study was approved by the Institutional Review Board of Seoul National University Hospital (IRB number: H2006-176-1135) and each participating institution. The requirement for informed consent was waived by the IRB of Seoul National University Hospital (IRB number: H2006-176-1135) and each participating institution, because of the retrospective nature of this study.

## Supplementary Information

Supplementary Information.
