# Supplementary material for: Early experience of laparoscopic resection and comparison with open surgery for gastric gastrointestinal stromal tumor: a multicenter retrospective study
Source: Sci Rep. 2022 Feb 10;12:2290. doi: 10.1038/s41598-022-05044-x (PMC8831629; doi:10.1038/s41598-022-05044-x)
Supplement: Supplementary file 1 — Supplementary Information. [file 41598_2022_5044_MOESM1_ESM.pdf]

Supplementary Figures and Tables

Early Experience of Laparoscopic Resection and Comparison with Open Surgery for Gastric Gastrointestinal Stromal Tumor: A Multicenter Retrospective Study

Journal Name: ***Scientific Report***

Shin-Hoo Park, MD[1], Hyuk-Joon Lee, PhD[1,2,3,*], Min-Chan Kim, PhD[4], Jeong-Hwan Yook, PhD[5], Tae-Sung Sohn, PhD[6], Woo-Jin Hyung, PhD[7], Seung-Wan Ryu, PhD[8], Yukinori Kurokawa, PhD[9], Young-Woo Kim, PhD[10], Sang-Uk Han, PhD[11], Hyung-Ho Kim, PhD[12], Do-Joong Park, PhD[1,2], Wook Kim, PhD[13], Sang-Il Lee, PhD[14], Haruhiko Cho, PhD[15, 16], Gyu-Seok Cho, PhD[17], Jin-Jo Kim, PhD[18], Ki-Han Kim, PhD[4], Moon-Won Yoo, PhD[5], Han-Kwang Yang, PhD[1,2,3]

[1]Department of Surgery, Seoul National University College of Medicine, Seoul, Korea
[2]Department of Surgery, Seoul National University Hospital, Seoul, Korea
[3]Cancer Research Institute, Seoul National University College of Medicine, Seoul, Korea
[4]Department of Surgery, Dong-A University College of Medicine, Busan, Korea
[5]Department of Surgery, University of Ulsan College of Medicine, Seoul, Korea
[6]Department of Surgery, Sungkyunkwan University School of Medicine, Samsung Medical Center, Seoul, Korea
[7]Department of Surgery, Yonsei University Health System, Yonsei University College of Medicine, Seoul, Korea
[8]Department of Surgery, Keimyung University School of Medicine, Seoul, Korea
[9]Department of Gastroenterological Surgery, Graduate School of Medicine, Osaka University, Osaka, Japan
[10]Center for Gastric Cancer, National Cancer Center, Seoul, Korea
[11]Department of Surgery, School of Medicine, Ajou University, Suwon, Korea
[12]Department of Surgery, Seoul National University Bundang Hospital, Seoul, Korea
[13]Department of Surgery, Yeouido St. Mary's Hospital, College of Medicine, The Catholic University of Korea, Seoul, Korea
[14]Department of Surgery, Chungnam National University Hospital, Seoul, Korea
[15]Department of Gastrointestinal Surgery, Kanagawa Cancer Center, Japan
[16]Department of Surgery, Tokyo Metropolitan Cancer and Infectious Diseases Center, Komagome Hospital.
[17]Department of Surgery, Soonchunhyang University College of Medicine, Seoul, Korea
[18]Division of Gastrointestinal Surgery, Department of Surgery, Incheon St. Mary's Hospital, The Catholic University of Korea, Seoul, Korea

Corresponding author: Hyuk-Joon Lee, M.D., Ph.D.
Department of Surgery, Seoul National University College of Medicine
Cancer Research Institute, Seoul National University College of Medicine
101 Daehak-ro, Jongno-gu, Seoul, 03080, Korea
Tel: +82-2-2072-1957
Fax: +82-2-766-3975
E-mail: appe98@snu.ac.kr

Running title: Laparoscopic surgery for gastric GIST

Supplementary figure 1. Bar graph of annually performed laparoscopic and open surgery for patients with gastric GIST between year 2000 and year 2007, before and after matching.

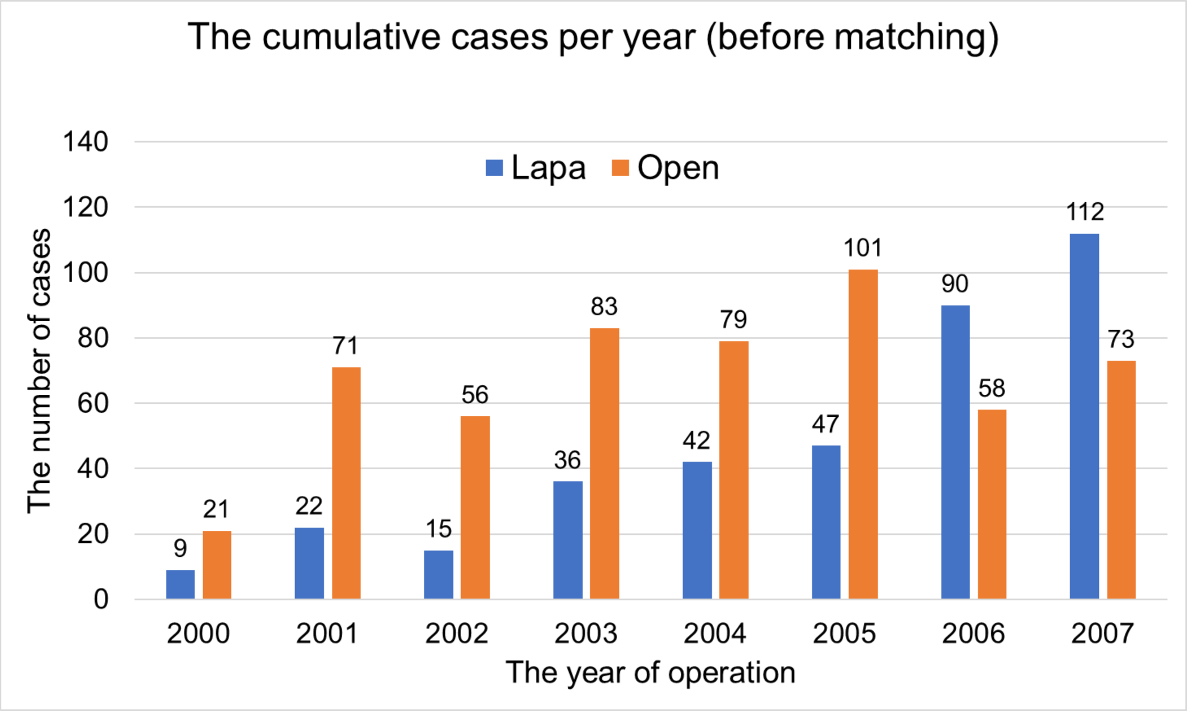

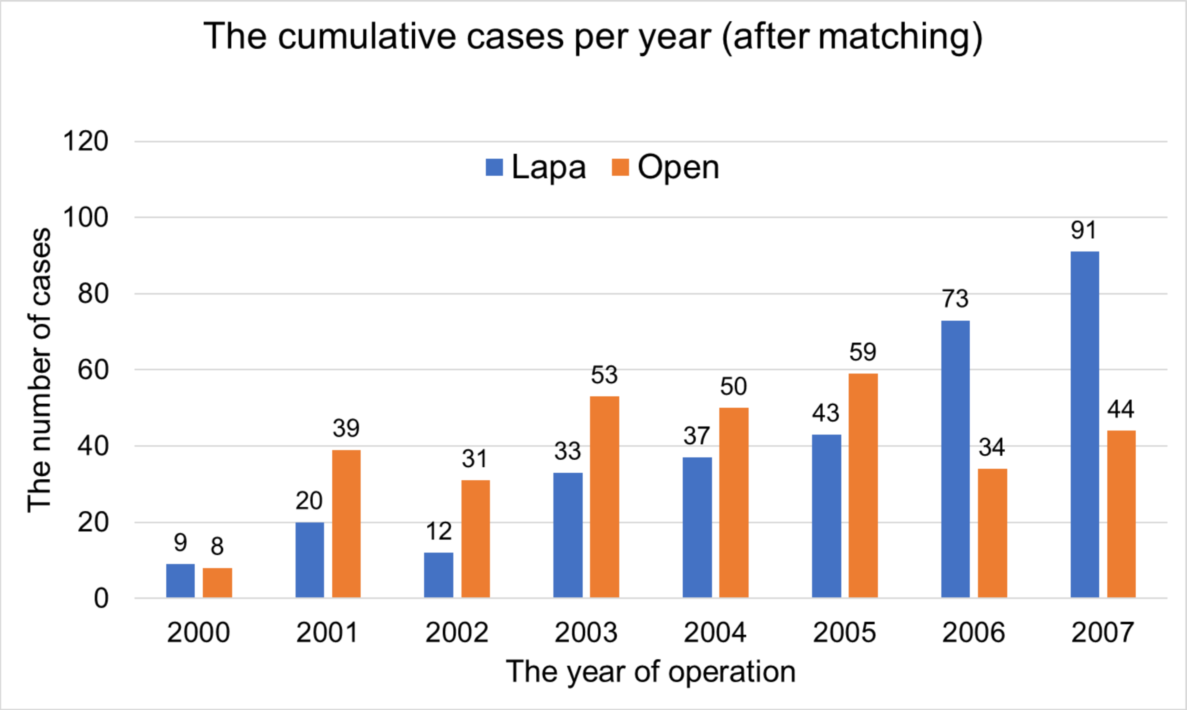

Supplementary figure 2.

a. Plot of absolute standardized mean differences of covariates before and after 1:1 propensity score matching.

b. Distribution of propensity scores in the laparoscopic versus open group before and after 1:1 score matching.

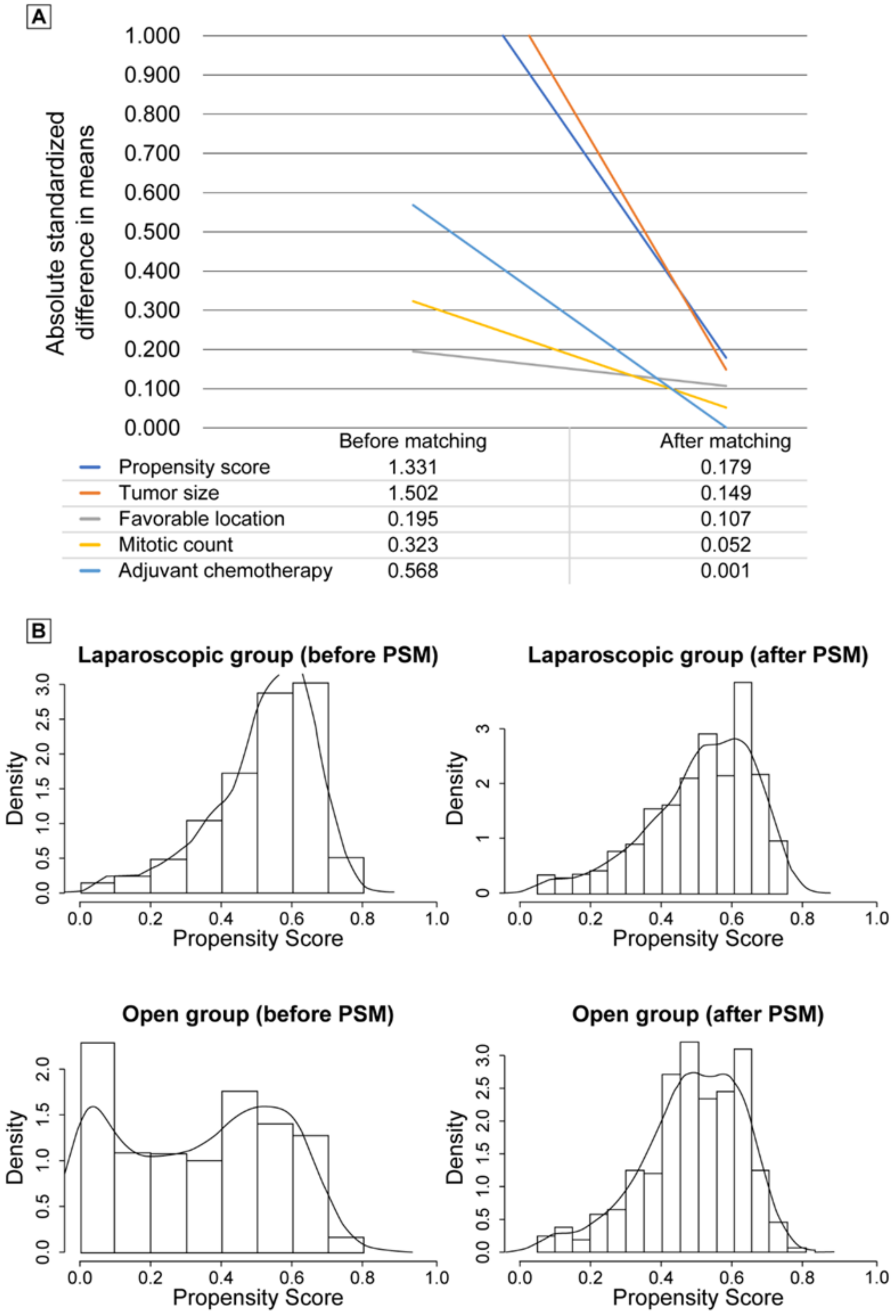

Supplementary Table 1. Cases that required conversion to open surgery during laparoscopic resection of gastric gastrointestinal stromal tumor.

					Location			
Case number	Age	Sex	BMI	Tumor size	Longitudinal	Circumferential	Preferences	Reason for conversion to open surgery
Case 1	59	F	22.8	70.0	Middle third	Lesser curvature	Unfavorable	Difficulty in handling large tumor with laparoscopic instrument
Case 2	74	M	26.0	30.0	Upper third	Posterior wall	Unfavorable	Difficulty in approaching the location of tumor
Case 3	69	F	36.0	32.0	Upper third	Greater curvature	Favorable	Severe adhesion due to previous abdominal surgery
Case 4	73	M	27.1	60.0	Upper third	Posterior wall	Unfavorable	Difficulty in handling large tumor with laparoscopic instrument
Case 5	46	F	24.6	80.0	GEJ to Cardia	Anterior wall	Unfavorable	Difficulty in handling large tumor with laparoscopic instrument<br>Difficulty in approaching the location of tumor
Case 6	37	M	25.0	130.0	Upper to middle	Greater curvature	Favorable	Difficulty in handling large tumor with laparoscopic instrument
Case 7	71	M	27.5	37.0	Middle third	Posterior wall	Unfavorable	Inflammatory adhesion to adjacent organs
Case 8	60	M	22.5	70.0	GEJ to Cardia	Lesser curvature	Unfavorable	Difficulty in handling large tumor with laparoscopic instrument<br>Difficulty in approaching the location of tumor
Case 9	77	M	27.0	75.0	Upper third	Greater curvature	Favorable	Difficulty in handling large tumor with laparoscopic instrument
Case 10	66	F	26.7	15.0	Lower third	Greater curvature	Favorable	Inflammatory adhesion to adjacent organs

Case 11	40	M	22.1	10.0	GEJ to Cardia	Posterior wall	Unfavorable	Difficulty in approaching the location of tumor
Case 12	54	F	18.2	35.0	Lower third	Anterior wall	Favorable	Inflammatory adhesion to adjacent organs
Case 13	63	M	27.5	38.0	GEJ to Cardia	Anterior wall	Unfavorable	Difficulty in approaching the location of tumor
Case 14	49	M	23.7	45.0	Upper third	Anterior wall	Favorable	Severe adhesion due to previous abdominal surgery

*TNM stage according to AJCC, the 7th edition.

Supplementary Table 2. Conversion to open surgery in laparoscopic resection

	Laparoscopic group before PSM (N = 373)		***P* value**	Laparoscopic group after PSM (N = 318)		***P* value**
**Tumor size (cm)**	Size ≤ 5 cm (N = 310)	Size > 5cm (N = 64)		Size ≤ 5 cm (N = 254)	Size > 5cm (N = 64)	
Conversion case (%)	8 (2.6)	6 (9.4)	0.020	5 (2.0)	6 (9.4)	0.011
**Locational preference**	Favorable (N=176)	Unfavorable (N=197)		Favorable (N = 145)	Unfavorable (N = 173)	
Conversion case (%)	7 (4.0)	7 (3.6)	0.830	3 (2.1)	8 (4.6)	0.356

Abbreviations: PSM propensity score matching

Supplementary Table 3. Univariate and multivariate logistic regression analysis for variables to predict the overall complications. Covariates were age, sex, body mass index (kg/m^2), underlying disease, year of operation, open resection, extent of resection, radicality, operation time, tumor size, locational preference, mitotic rate, NIH risk classification.

Variables	Univariate logistic regression			Multivariate logistic regression		
	B	Odds ratio (95% CI)	*P* value	B	Odds ratio (95% CI)	*P* value
**Age, years per increase**	-0.005	0.995 (0.966-1.025)	0.732			
**Male** (vs. female)	0.370	1.448 (0.732-2.848)	0.284			
**Body mass index** (kg/m^2, per increase)	0.080	1.084 (0.974-1.206)	0.142			
**Underlying disease**	0.052	1.053 (0.535-2.073)	0.881			
**Year of operation**						
Before 2006 vs. from 2006	-0.038	0.963 (0.483-1.920)	0.915			
**Open** (vs. laparoscopic) **resection**	0.868	2.381 (1.151-4.927)	0.019	0.938	2.555 (1.195-5.463)	0.016
**Extent of resection**						
gastrectomy vs. wedge or enucleation	-1.090	0.336 (0.045-2.509)	0.288			
**Radicality, R1** (vs. R0 resection)	0.629	1.876 (0.231-15.229)	0.556			
**Operation time** (minutes, per increase)	0.009	1.009 (1.004-1.014)	<0.001	0.010	1.100 (1.005-1.015)	<0.001

**Tumor size**						
>5 cm (vs. ≤5 cm)	0.893	2.443 (1.215-4.912)	0.012	0.983	2.627 (1.280-5.575)	0.009
**Locational preference**						
Unfavorable vs. favorable	0.721	2.057 (0.975-4.341)	0.058	0.966	2.627 (1.164-5.927)	0.020
**Mitotic rate (per 50 HPF)**						
>5, ≤10 (vs. ≤5)	0.056	1.058 (0.425-2.632)	0.904			
>10 (vs. ≤5)	0.077	1.080 (0.316-3.696)	0.902			
**NIH risk classification**						
High (vs. very low to intermediate)	0.480	1.615 (0.684-3.814)	0.274			

Abbreviations: CI confidence interval; HPF high power field; NIH National Institutes of Health
